# The Impacts on Health, Society, and Economy of SARS and H7N9 Outbreaks in China: A Case Comparison Study

**DOI:** 10.1155/2018/2710185

**Published:** 2018-06-28

**Authors:** Wuqi Qiu, Cordia Chu, Ayan Mao, Jing Wu

**Affiliations:** ^1^Dr., Director, Department of Public Health Information Research, Institute of Medical Information, Chinese Academy of Medical Sciences, 3 Yabao Road, Chaoyang District, Beijing 100020, China; ^2^Dr., Professor, Director, Centre for Environment and Population Health, Griffith University, 170 Kessels Road, Nathan, Brisbane, Queensland 4111, Australia; ^3^MPH, Associate Professor, Department of Public Health Information Research, Institute of Medical Information, Chinese Academy of Medical Sciences, 3 Yabao Road, Chaoyang District, Beijing 100020, China; ^4^Vice Director, Chinese Centre for Health Education, 12 Anhua Xili 1 Qu, Anding men Wai, Chaoyangqu, Beijing 100011, China

## Abstract

**Background:**

Epidemics such as SARS and H7N9 have caused huge negative impacts on population health and the economy in China.

**Aims:**

This article discusses the impacts of SARS in 2003 and H7N9 in 2013 in China, in order to provide a better understanding to government and practitioners of why improving management of response to infectious disease outbreaks is so critical for a country's economy, its society, and its place in the global community.

**Methods:**

To provide the results of an analysis of impacts of SARS and H7N9 based on feedback from documents, informants, and focus groups on events during the SARS and H7N9 outbreaks.

**Results:**

Both outbreaks of SARS and H7N9 have had an impact on China, causing significant negative impacts on health, the economy, and even national and even international security.

**Conclusions:**

Both SARS coronavirus and H7N9 viruses presented a global epidemic threat, but the social and economic impacts of H7N9 were not as serious as in the case of SARS because the response to H7N9 was more effective.

## 1. Introduction

In the past 15 years China has experienced numerous public health crises caused by disease outbreaks including Severe Acute Respiratory Syndromes (SARS) in 2003 and Influenza A Virus Subtype H7N9 (H7N9) in 2013. Epidemics such as SARS and H7N9 have caused huge negative impacts on population health and the economy. If not controlled well, they can become pandemics, threatening national and even international security. SARS, in particular, highlighted global connectedness and the great threat that pandemic and potential pandemic present.

Since the SARS outbreak in 2003, China has established and strengthened its national and local surveillance systems to prevent and control diseases and has also expanded its laboratory capacity [1, 2]. China's experiences of emergency management for epidemics have varied. Although the SARS coronavirus and H7N9 virus share some similarities, the control efforts for SARS were problematic and the disease spread globally [3], while the H7N9 response was highly praised and the disease did not spread widely [4]. This article discusses the impacts of SARS in 2003 and H7N9 in 2013 in China, in order to provide a better understanding to government and practitioners of why improving management of response to infectious disease outbreaks is so critical for a country's economy, its society, and its place in the global community.

## 2. Methods

We followed the methods of Qiu W. et al., 2017 [5]. This research uses a qualitative case study approach including literature review, document analysis, and in-depth interviews.

The review drew on a wide range of data sources, including books, journal articles, government documents, policy reports, and conference papers. Most books were searched for in the Griffith University Library Catalog. Journal article searches were made in the Library Catalog and reference lists of retrieved articles and textbooks, and electronic literature databases, such as ScienceDirect, PubMed, Medline, Health and Medical Complete (ProQuest), and Web of Science. Government documents and policy reports came from the national and local Centers for Disease Control and Prevention (CDC), government departments, and published research literature.

In-depth interviews using a semistructured style were conducted with 26 key stakeholders, including officers from various national and international agencies as well as experts from local health departments, agriculture departments, Centers for Disease Control and Prevention (CDCs), hospitals, and journalists who have experience of SARS and/or H7N9 in the key cities of Beijing, Shanghai, Guangzhou, and Hangzhou, which were most affected by SARS and/or H7N9. We interviewed key informants about their experience of and reflections on the emergency management of the SARS and H7N9 events, with the same questions about the impacts on health, society, and economy of SARS and H7N9. Each interview lasted about 60 minutes.

## 3. Results

### 3.1. Impacts of SARS in China in 2003

#### 3.1.1. Health Effects

The SARS outbreak infected thousands of people, causing widespread serious illness across a large population and many deaths. According to WHO, from Nov 1, 2002, to July 31, 2003, 648 of the 8082 probable cases of SARS in mainland China and Hong Kong died. Worldwide, in just 6 months, there were more than 8000 infected individuals, with over 700 deaths (almost 9% of infected cases) [6]. The psychological impact of SARS was also very serious. The distress was more prominent among the groups of nurses who were working with patients with SARS [7]. Studies show that the SARS outbreak also fostered negative impacts on people's mental health [8], as mentioned by two hospital doctors:

These SARS cases caused extreme emotional sadness. Psychologically it is entirely possible that an event destroyed a person. They needed psychological counselling.

When the SARS cases lived in the hospital, they could not see their family, and feared the treatment. They developed a mental disorder.

#### 3.1.2. Social Impacts

SARS caused a very large impact on society, particularly in China. During the early period of the SARS outbreak, tension surged in the community. Due to a lack of trustworthy official information, folk tales about the epidemic situation spread through word of mouth, mobile phone short messages, social media transmission, and other ways. The spread of all kinds of rumors exacerbated the spread of social panic, reflected in an escalation of panic buying of drugs in Guangdong province [9]. One rumor was that Banlangen (*Radix isatidis*) and vinegar could prevent and control SARS, but whether they were effective for SARS was not scientifically established at this time. In early January 2003, the first wave to purchase antiviral drugs occurred in Heyuan city. After half a month, the drug purchasing spree had spread to Zhongshan city; then the buying spree gradually spread through Guangdong province [10, 11], as mentioned by a community resident:

Everybody was panic buying Banlangen (Radix isatidis). Banlangen was completely sold out.

In February 2003, people were wearing masks everywhere on the streets in Guangzhou. Panic was also spreading from Guangzhou to Shenzhen, Zhuhai, and other areas and then spread to Hainan, Fujian, Jiangxi, Guangxi, Hong Kong, and other adjacent areas. A media journalist said the following:

During SARS, we were more likely to panic. I had the impression that Banlangen (Radix isatidis) was sold out. Like every family, I also went to buy Banlangen (Radix isatidis) and vinegar, which they thought can cure SARS. Now I think that was a very funny thing to do.

By the middle of March, because the epidemic was spreading but no information had been officially confirmed, people began to believe the rumors, and the panic and purchasing of antiviral drugs that had appeared in Guangdong also began in Beijing, as mentioned by an officer of international organization:

During SARS, I was working in a unit outside of Beijing. Beijing was in a panic. When I arrived at Beijing, (my colleagues) gave me a box of masks and they made me wear a mask. To tell the truth, I felt a bit nervous.

The lack of understanding of SARS by authorities or the media caused a number of experts to become dissatisfied. For example, a 72-year-old retired surgeon from the People's Liberation Army 301 Hospital, wrote to the media criticizing the health department for hiding the SARS epidemic situation. On April 12, he also wrote a letter to the MOH, urging them to publish accurate numbers as soon as possible. On the same day, an academic from the Chinese Academy of Engineering, the leader of the team guiding the prevention and cure of SARS in Guangdong province, also questioned the information provided by government about the control of the epidemic. He questioned whether SARS really was under control. These published questions brought the SARS epidemic situation in China to the attention of the international community [12].

#### 3.1.3. The Economic Impacts

The SARS epidemic brought great harm not only to peoples' physical and mental health, but also to the economy. It was estimated that Asian states lost USD 12–18 billion as the SARS crisis depressed travel, tourism, and retail sales [13]. SARS had a large impact on tourism and its related industries, and due to the spread of SARS, population movement in China and many counties decreased. Families reduced their demand for food, clothes, travel, and entertainment, and the numbers of guests in hotels declined sharply. As observed by officers from the Agricultural and Health Departments,

I think it was certainly panic at beginning, as it was not clear what SARS was. I remember (there were) almost no people in a restaurant when I had dinner. And the tourism had few people too.

During SARS in Shanghai, there were not many people on the street and almost no people in entertainment clubs, restaurants and gymnasiums, which caused a very large impact on the whole social and economic life.

After WHO announced that Beijing was an epidemic area and issued more stringent advice to international travelers and airlines, including recommendations on screening at certain airports, the international tourism, transport, and business sectors were seriously affected. For example, the mid-April Chinese enterprise summit in Beijing, hosted by the World Economic Forum, was delayed and the Rolling Stones concert planned for Beijing was cancelled.As observed by an international officer and a media journalist,

During SARS, it was very obvious to see the status of Beijing which became a ghost city. We all know that Beijing has traffic jams every day, but [then] you worried whether you were speeding. It's never been seen before.

During SARS, you could find that Beijing traffic was so good, (there were) not many people on the road. There were no traffic jams, and you felt great to take the bus (with few people) in Beijing. But I was deeply impressed that when I took a bus, and a man behind had a cough, I was scared and I got off quickly at the next stop.

The global macroeconomic impact of SARS was estimated at USD 30–100 billion or around USD 3–10 million per cases [14]. The 2003 SARS outbreak caused losses of USD 12.3-28.4 billion and an estimated decrease of 1% in GDP in China and 0.5% in Southeast Asia [15]. The social burden of SARS in Guangzhou meant less income and spending, with a rough estimate of the total economic burden of RMB 11 billion [16].

The influence of SARS also spread to the manufacturing industry. It was reported that in Asia's largest manufacturing base, Dongguan in Guangdong province, because of the reduced orders from Hong Kong, the shipments from Dongguan to Hong Kong decreased by one-third [17].

At the same time personnel exchanges were reduced for fear of infection, and income decreased. There was also increased spending on prevention and healthcare, which had negative economic impacts on families. Interviews with 71 households in Qinling Mountain in Shaanxi Province indicated that in the second quarter of 2003 SARS caused the average annual household income to decline to US$175.44, 22.36% below what was expected [18].

### 3.2. Impacts of H7N9 in China in 2013

#### 3.2.1. Health Effects

H7N9 avian influenza is another infectious disease that has caused severe illness and death in humans in China. It has a high fatality rate [19]. The first H7N9 case was found in China in February 2013. By November 13, 2015, a total of 681 laboratory-confirmed cases of human infection with H7N9, including 275 deaths were reported to WHO. The case fatality rate of H7N9 was 40.1% [20]. According to Disease Outbreak News issued by the WHO on February 22, 2017, a total of 1223 laboratory-confirmed cases of human infection with avian influenza A (H7N9) virus had been reported since early 2013. The number of human cases developing since October 1, 2016, accounted for nearly one-third of all human cases of H7N9 infection reported since 2013. As of February 23, 2017, at least 425 cases had been reported during the fifth outbreak in China, which began in October and spiked suddenly in December in 2016. This increase in the number of new cases of H7N9 infection has caused domestic and international concern [21]. According to National Statutory Epidemic Situation in 2017 by the China National Health Commission on February 26, 2018, there are 589 laboratory-confirmed cases of H7N9 that had been reported, with 259 deaths in China in 2017. Today, there is no H7N9 vaccine available, although some vaccine manufacturers are conducting clinical evaluations of a H7N9 vaccine [22].

The influenza H7N9 virus remains a large threat due to its virulent nature in poultry. The major factors that influence the epidemic potential of an influenza virus, including its ability to cause human disease, are the immunity of the population to the virus and the transmission potential of the virus [23]. Although there is no evidence that H7N9 spreads easily from human to human and the population had little immunity to H7N9, the virus was easily transmitted. The significance still remains over whether H7N9 could be the next pandemic strain of influenza [24].

#### 3.2.2. Social Impacts

Although there were rumors that people could be infected with H7N9 from eating chicken and that pickled peppers and onions can prevent H7N9 [25], compared with SARS, the H7N9 epidemic did not lead to large-scale social panic, and the management of the problem satisfied both the Chinese and international community, as mentioned by one media journalist and one CDC expert

During H7N9, the impact on people's lives was very limited. In fact, the panic is derived from what people don't know. There wasn't any panic, as we knew something with H7N9.

There were no impacts on the city life in Beijing during H7N9. The only [impacts] was to further strengthen the poultry market management.

The National 12320 Telephone Management Center carried out an opinion survey regarding the government's response to the H7N9 event from April 27 to May 4 through the 12320 Health Hotline, which was reported in* Guangming Daily *in May 2013. In it, more than 80% of respondents expressed satisfaction with the government's prevention and control of human infection with H7N9 avian influenza, thought that the government announced the information regarding the epidemic situation in a timely manner, expressed satisfaction with the government's release of information about prevention and control measures, and felt confident in the government's ability to fully control the epidemic, as mentioned by a community resident:

As we could know the information of H7N9 by TV, newspaper, internet, it was clearer for us to know the dangers of H7N9 than SARS.

More than 50% of the respondents believed that the prevention of human infection with H7N9 avian influenza had changed their health habits, indicating that the release of the knowledge of prevention and control of human infection with H7N9 avian influenza was effective [26].

**Figure 1:**
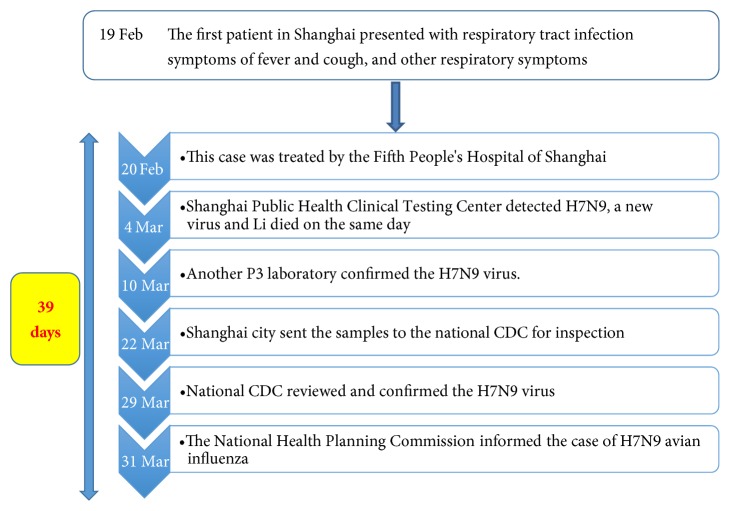
Timeline from first hospitalization of a case to confirmation and notification of H7N9 in 2013.

The timeline for the beginning of the outbreak of H7N9 is presented in Figure 1.

From this figure, we can draw a conclusion that the communication strategy of the Chinese Government in dealing with H7N9 was very successful, as identified by an officer of an international organization:

I knew the characteristics of H7N9. The government management of the health and agriculture sectors was completely open, so I completely believed them and I felt no panic. There wasn't any influence on my personal life during H7N9.

Comparing the H7N9 avian influenza and the SARS crisis, most interviewees thought that SARS was more serious and life threatening. Because SARS was characterized by person-to-person transmission and at that time the government did not have the system or experience to deal with public health emergencies, the disease was transmitted quickly and social panic ensued. The fast and effective countermeasures by Chinese authorities to H7N9 avian influenza were not only highly considered by the inspection mission of WHO and World Organization for Animals, but also presented a model to the world. The Associated Press (an American news agency) praised “China's new openness to deal with bird flu and cooperation with international organizations”;* Nature* claimed in an editorial entitled “The Battle against Bird Flu” that “Currently, China reports on the epidemic every day and the media discussion is also quite open and frank” [4]. China research personnel cooperated with their counterparts across the world and published detailed analyses of the virus quickly in academic journals.

The H7N9 outbreak also became a food culture issue. The closure of live poultry markets (LPMs) caused some changes in the Chinese food culture of eating freshly killed chickens, as mentioned by a community resident:

Most families did not buy live poultry and were careful about their health habits during H7N9.

#### 3.2.3. The Economic Impacts

The economic impacts of H7N9 were less serious than SARS but still important to characterize. Studies show that the direct medical costs of hospitalization of a patient with H7N9 were estimated to be RMB 71 060, which is more than a year's income for a person in a rich province in China [20]. In April 2013, the H7N9 avian influenza epidemic caused the price index of meat and poultry and their products to fall to 101.5 on a year-on-year basis. As a result of the outbreak, China's poultry industry suffered a loss of more than RMB 40 billion [27]. However the economic impact was little in the global community.

The high mortality of H7N9 changed the attitude of the public towards chickens and it became apparent that few cared for chickens in the market. Many places closed their live poultry trading, while the virus resulted in serious economic losses to farmers. At the same time, consumers' confidence in poultry products declined, which had an important influence on meat and poultry prices, as mentioned by a CDC expert:

During H7N9, like everyone else, the consumption of poultry was indeed reduced in my family.

Overall, the health, societal, and economic impacts of the two infectious disease outbreaks were quite different as summarized in Table 1.

## 4. Discussions and Conclusion

The outbreaks of SARS and H7N9 represented serious public health emergency crisis events in China, and both had significant impacts on health, society, and the economy.

Both virus had not been reported in human beings previously. They both can lead to severe disease, characterized by high fever, severe respiratory symptoms, and death, and there are still no specific antiviral drugs and vaccines for them. SARS coronavirus is thought to be an animal virus arising from an as-yet-unknown animal reservoir (perhaps bats) that spread to other animals (civet cats) and then to the first infected humans in southern China in 2002. For the H7N9 virus, the animal reservoir is poultry. Worldwide, people of all ages had little protective immunity and both viruses presented a global epidemic threat [28, 29].

China's emergency management of the two epidemics varied. After SARS, China's public health emergency management system developed very fast, and as a consequence emergency management greatly improved [30]. Despite the similarities of SARS and H7N9 and the fact that mortality of H7N9 was much higher than SARS, control efforts for SARS were slow to be mobilized and were heavily criticized and generally considered to be suboptimal, as the poor handling of SARS exposed serious information communication problems in the then emergency management system processes. In contrast, although the H7N9 has not been identified as a pandemic in China as there is limited person-to-person spread of H7N9 under specific circumstances such as poultry handling, the Chinese Government's response to H7N9 was much swifter and more transparent than it was in the SARS outbreak. Consequently, the social and economic impacts of H7N9 were not as serious as in the case of SARS. This points to the evolution of the emergency management system, highlighting how a transparent and rapid response can reduce the impacts of infectious disease outbreaks.

An effective and efficient emergency response can reduce avoidable mortality and morbidity and reduce the economic, social, and security impacts of all public health emergencies including disease outbreaks [31]. Understanding risk communication practice is an important element in understanding the different management responses to SARS and H7N9 and subsequent outcomes. The effectiveness of emergency preparedness and responses is highly dependent on the quality and amount of information that is available at any given time, and quality communication and coordination among partners is crucial. Information sharing and communication are considered key tools for the coordination of prevention and management of infectious diseases.

## Figures and Tables

**Table 1:** Impact on health, society, and economy of SARS and H7N9 in China.

**Categories**	**SARS**	**H7N9**
**Health effects**	(i) In 2003 in China: 5327 cases, 349 deaths; mortality rate 6.6% (ii) By 11 July 2003, the virus had spread to 29 countries and regions, with a cumulative number of confirmed cases of 8096 people, 774 people deaths and an average death rate of 9.6%.	(i) In 2013 in China: 135 cases, 45 deaths; mortality rate 33.6% (ii) By December 3, 2013, a total of 148 cases of H7N9 avian influenza were confirmed on the Chinese mainland, Taiwan and Hong Kong area, where 48 died, with a case fatality rate of 32.43%

**Social impacts**	**Panic, criticized** (i) Information was “doctored” and delayed. (ii) Rumors and social chaos (iii) Food, salt and Banlangen (Radix Isatidis) were sold out (iv) Flights were cancelled (v) Schools were closed (vi) large mass-gathering events cancelled	**Social stability, praised** (i) Reliable information, promptly released (ii) No social chaos (iii) The management of the problem satisfied both the Chinese and international community.

**Economic impacts**	(i) The global macroeconomic impact of SARS was estimated at USD 30–100 billion or around USD 3–10 million per case (ii) Caused losses of USD 12.3-28.4 billion and an estimated decrease of 1% in GDP in China	(i) China's poultry industry suffered a loss of more than 40 billion RMB (ii) There was little economic impact in the global community

## Data Availability

The datasets generated and/or analyzed in this study are available from the first author or corresponding author on reasonable request.
